# The fecal resistome of beef cattle from conventional grain-fed and grass-fed systems in the Western United States

**DOI:** 10.1186/s12866-025-04562-8

**Published:** 2025-11-28

**Authors:** Yuyuan Feng, Frederick Yang, Sarah C. Klopatek, James W. Oltjen, Xiang Yang

**Affiliations:** 1 Department of Animal Science, University of California, Davis, CA 95618 USA; 2 Nelson Institute for Environmental Studies, University of Wisconsin- Madison, Madison, WI 95616 USA

**Keywords:** Cattle, Resistome, Grass-fed, Antimicrobial resistance, Meat safety

## Abstract

Bacteria in the gastrointestinal tract of cattle may develop antimicrobial resistance (AMR) due to the use of antibiotics in live animals and can be excreted in feces, posing a risk of contamination. However, it remains unclear whether different beef production systems influence the levels of AMR in cattle feces. The objective of this study was to characterize and compare the fecal resistome of cattle raised in grass and grain-feeding systems in the Western United States. Fecal samples were collected from individual cattle at 14 months of age and two days before their respective harvest date. Groups included: (1) Conventional grain-fed (CON, *n* = 10), (2) Grass-fed for 20 months (20GF, *n* = 10), (3) Grass-fed and then grain-finished for 45 days (GR45, *n* = 10), (4) Grass-fed for 25 months (25GF, *n* = 10). According to metagenomic analysis, grass-feeding systems, particularly the one with extended grass-feeding, are associated with a less diverse resistome. The 25GF group had smaller *(P* < 0.05*)* Chao1 value than the other groups at the harvest time. Antimicrobial resistance genes (ARGs) richness and evenness were higher in CON and GR45 than in 20GF and 25GF (*P* < 0.05). Additionally, the resistome of GR45 and CON differed from 25GF (*P* = 0.018). In grass-feeding systems where antibiotics were not administered, animals’ feces exhibited greater *(P* < 0.05*)* diversity in transferable biocide and metal resistant genes (BMRGs) compared with the grass-fed but grain-finished system. Greater ARG diversity in grain-finished feeding systems may enhance the spread of antimicrobial-resistant bacteria (ARB) during production, posing additional risks to food safety. Similarly, higher BMRG diversity observed in grass-fed systems may promote ARB spreading through co-selection mechanisms, which could also contribute to potential food safety concerns.

## Background

The Centers for Disease Control and Prevention [1] reported that antimicrobial resistance (AMR) is responsible for over 2 million infections and 23,000 deaths annually. Furthermore, the Organization for Economic Co-operation and Development has predicted that between 2015 and 2050, AMR will cause 30,000 deaths in the United States. Consequently, it is crucial to address the development of AMR and spread of antimicrobial resistant genes (ARGs) to mitigate their impact on public health. The widespread use of antibiotics in livestock industries for disease control and enhancing feed efficiency has been a long-standing practice [2]. However, the latter purpose was prohibited by the United States Food and Drug Administration (FDA) in 2017 [3]. In the United States, the largest beef-producing nation, critically important human antibiotics such as fluoroquinolones, macrolides, and third-generation cephalosporins are commonly utilized in beef production to prevent or treat disease [4, 5]. Research has demonstrated that the routine administration of antibiotics in livestock may contribute to the selective pressure for bacteria to develop AMR, which facilitates the transmission of AMR to humans through the consumption of fecal-contaminated food and leads to decreased antibiotic effectiveness for treating infections in humans [6–8].

Recently, there has been a notable surge in consumer awareness regarding food safety, leading to a greater willingness to invest in food products perceived as safer [9–11]. Beef constitutes a widely consumed source of protein for humans, exhibiting a steady growth in global demand [4]. The United States accounts for the highest consumption of beef, particularly grain-fed beef, worldwide [12]. However, recent data indicates that approximately 40% of consumers perceive grass-fed beef (cattle fed 100% grass from weaning to harvest) as safer than conventional grain-fed beef (cattle that have been finished in a feedyard for over 100 days) [12]. Factors such as animal welfare, environmental sustainability, and health considerations contribute to an increasing consumer preference for grass-fed beef, with some consumers demonstrating a willingness to pay a premium for these products [12, 13]. Grass-feeding systems strictly prohibit the utilization of grains and grain byproducts [14], and, although not explicitly specified by the USDA, many who market grass-fed beef avoid using antibiotics or hormones [15].

Conventional grain-based feeding systems, where animals are finished in feedlots, and grass-feeding systems exhibit differences in diet and in antibiotic usage. However, it remains uncertain whether different feeding systems employed in beef cattle production led to an influence on the resistome, which is defined as the density and composition of ARGs representing the AMR potential [16]. Previous research has yielded varying results concerning the differences in resistant bacteria or resistance genes arising from distinct cattle operations [17–20]. However, early research primarily focused on analysis of the prevalence data [17] or the specific bacteria [18] that exhibited the expressed ARGs at phenotypical level. These approaches cannot capture the overall picture of the antimicrobial resistance landscape, as they ignore the interactions between bacteria or the interactions between bacteria and their environment. Thus, metagenomics, which involves sequencing the whole DNA content from a given sample, has recently been introduced as a better approach to analyze the resistome from ecological perspective [21, 22].

In the Western United States, particularly in California, the number of beef cattle grew, with a recorded population of more than 1.6 million in 2021, a 1.1% increase from the previous year [23], which implied that demand for grass-fed beef in this region had also risen. Consequently, it is crucial to examine how beef cattle feeding systems impact meat safety in this area. Previous studies have primarily focused on characterizing the profile of AMR in cattle raised with or without antibiotics within the same feeding system. However, some studies show that antimicrobial use is not directly linked to changes in microbial resistance, and thus other factors must be taken into consideration [17, 20, 24, 25]. The potential confounders such as diet composition, environment, and cattle age could also influence the fecal resistome. Studying these systems enables the identification of complex relationships and interactions by considering factors such as age, animal intensity, and environmental conditions. Thus, as the first to investigate AMR in cattle from both grain and grass-feeding systems, our study aimed to characterize and compare the fecal resistome of cattle raised in different grass and grain-feeding systems currently in use within the Western United States.

## Methods

### Sample collection

This project was conducted following protocol approved by the Institutional Animal Care and Committee at the University of California Davis (UCD; protocol #20560). Fecal samples were collected from animals used by Klopatek et al. [26]. In June 2018, forty Angus and Angus-Hereford cross beef steers were fenceline weaned at the University of California Sierra Foothill Research and Extension Center (Browns Valley, CA). In total, 68 cattle (average initial weight of 284 kg ± 27.57 kg) were kept in an animal trial done by Klopatek et al. [26], and a subset of 40 cattle was selected for inclusion in the present study. At each sampling point, 10 fecal samples were collected from steers that were randomly selected from each group (4 groups) to characterize the fecal resistome using metagenomic sequencing. The four groups were: (1) Conventional grain-fed (CON), (2) Grass-fed for 20 months (20GF,), (3) Grass-fed and then grain-finished for 45 days (GR45,), and (4) Grass-fed for 25 months (25GF). All steers grazed on pasture in Maxwell, CA from June to November 2018. Afterward, CON steers were moved to the UCD feedlot and fed a starter ration for 14 days, followed by an intermediate ration for 14 days, and finally finished on a high-energy corn-based ration for 100 days. Meanwhile, 20GF and 25GF steers were transported to the Sierra Field Research Station in Browns Valley, CA, where they were provided a mixture of grasses.

The 20GF steers were harvested at the end of the winter-spring grazing season, while all GR45 cattle were those diagnosed with pinkeye infection and treated with antibiotics and then at the end of the winter-spring grazing season moved to the UCD feedlot and fed a starter ration for 7 days, followed by an intermediate ration for 10 days, and finally finished on a high-energy corn-based ration for 45 days. At the end of the winter-spring grazing season, the 25GF steers were transported to the UCD flood-irrigated pasture in Davis, CA, where they were given a mixture of perennial grasses. Cattle in the 20GF and 25GF groups were never given antibiotics or ionophores. Conversely, Monensin was included in the ration for CON and GR45 steers. Additionally, the one-time injection of oxytetracycline and florfenicol were administered to CON (2 of 10) and GR45 (10 out of 10) steers who presented the symptoms for pinkeye, liver abscess, or pneumonia. Minerals were added to the animals’ feed at the University of California Sierra Foothill Research and Extension Center and the UCD feedlot. The CON and GR45 steers were harvested at a large-scale commercial processing plant in Fresno, CA, while the 20GF and 25GF steers were harvested at a natural/organic beef packing plant in Merced, CA.

This study was a longitudinal study. Rectal fecal samples were collected from each steer as a baseline (10 from each group) before they were assigned to a group, and again one week before harvest (10 from each group). Approximately 50 g of fecal samples were collected per sampling time from each individual and stored in separate sterile 24 oz Whirl-Pak sampling bags (Whirl-Pak, Madison, WI). The samples were then transported to UCD within two hours and stored at −80 °C for further analysis.

### Sample processing and DNA extraction

The fecal samples stored at −80 °C were moved to 4 °C to thaw overnight. Total microbial DNA was extracted from the fecal samples using the DNeasy PowerSoil Pro Kit (Qiagen, Valencia, CA), following the manufacturer’s protocol. The concentration of the extracted DNA was determined by measuring the absorbance at 260 nm using the Invitrogen Qubit Fluorometer (Thermo Fisher Scientific, Inc, Pittsburgh, PA). The purity of the extracted DNA was assessed using the NanoDrop spectrophotometer (Thermo Fisher Scientific, Inc, Pittsburgh, PA). For an accepted concentration, the 260/280 and 260/230 ratios were > = 20ng/µL, 1.8–2.8, and > 2, respectively. For samples with low concentration, QIAquick PCR Purification Kit was used (Qiagen, Valencia, CA) following the manufacturer’s protocol to increase the concentration of the samples.

### Library preparation and sequencing

The extracted DNA samples were sent to UC Davis Genomic Center for library preparation and metagenomic sequencing. The sample libraries were built by using the Illumina TruSeq DNA library kit (Illumina, Inc, San Diego, CA). Library sequencing (paired-end, 2 × 150 bp) was performed on the Illumina NovaSeq 6000 in the Genome Center at the University of California, Davis.

### Bioinformatics

The sequencing reads were processed by first trimming and merging them using HTStream (version 1.3.3) (https://s4hts.github.io/HTStream/). Reads with a quality score ≤ 15 were removed, and sequences shorter than 36 bp after trimming were discarded to ensure high-quality downstream analysis. Then the steps and results were visualized by MultiQC (version 1.14) [27]. BWA (version 0.7.12) [28] was used to filter out host DNAs (Bos Taurus, UMD3.1). The filtered reads were then matched to the MEGARes (version 2.0) [29], which classifies the antimicrobial drug, biocide, and metal resistance factors in metagenomic sequencing data, using BWA with the default settings, and ARGs were identified at gene, mechanism, and class levels, using ResistomeAnalyzer (https://github.com/cdeanj/resistomeanalyzer). Next, the identified ARG reads were assembled into contigs using MEGAHIT (version 1.2.9) [30], and BWA-MEM [28] was used to align the contigs that contained ARG reads for identifying bacterial origin of these contigs. The unassembled reads that were identified as ARGs were also classified to a customized database including RUG2 [31] FASTA sequences, which contains the extensive amount of assembled rumen genomes to identify the potential bacteria origin of the ARGs at species level, and RefSeq [32] FASTA sequences, which contains sequences from more than 55,000 organisms for better taxonomic representation, using Kaiju (version 1.9) [33]. The contigs were compared with the reference genome in Kaiju database for getting the number of reads that were assigned to each taxon. The merged reads were aligned to Resfinder (version 4.0) [34] with a 90% identity threshold and 60% minimum length match for identifying transferable ARGs. Additionally, the host-removed, non-merged reads were aligned to the BacMet database with experimentally confirmed resistant genes (version 2.0) [35] using DIAMOND (version 2.0.15) [36] with an E-value cutoff of ≤ 10^− 10^ to identify the biocide and metal resistant genes (BMRGs).

### Statistical Analysis

The resistome was analyzed based on the relative abundance of ARGs and BMRGs, which was calculated using the number of reads of each gene/class/mechanism divided by the total reads present in this sample. To normalize the relative abundance, the cumulative sum scaling (CSS) method was used using metagenomeSeq R package (*v1.42.0*) [37] with default settings. Shannon index (representing richness and evenness) and Chao1 index (representing richness) of ARGs and BMRGs were calculated using the vegan package (*v2.6-4*) [38] and then were utilized to analyze alpha diversity. Beta diversity of ARGs and BMRGs was represented by Non-Metric Multidimensional Scaling (NMDS) using Bray–Curtis dissimilarity calculated through the vegan package. Multivariate homogeneity of groups variances following PERMANOVA with 999 permutations was examined using the vegan package. To identify changes in relative abundance from baseline to harvest within each feeding system, the mean relative abundance of each ARG was calculated for all groups. To ensure valid data, a value of one was added to each data point in the data frame, and log2 fold change was applied to the modified mean of normalized relative abundance between harvest and baseline for each group at the class level.

The completely randomized design was used to examine the feeding system effects and age effects on the resistome. Feeding system effects were defined as the difference between grass-feeding systems and grain-feeding systems at harvest, while age effects were defined as the difference between baseline and harvest for each feeding system. Accordingly, a two-way analysis of variance (ANOVA) with Tukey test were applied to explore the feeding effects, age effects, and corresponding interaction effects on the alpha diversity. Pairwise comparisons were done on the top 10 classes of ARGs between groups using t-test. The P-values obtained from multiple comparisons were adjusted by the Bonferroni method. In addition, the feeding effects on the microbiome were tested by one-way ANOVA. All statistical analyses and visualization were conducted in R statistical software (version 4.1.2), with an alpha level of 0.05 applied to test statistical significance.

The relative abundance data of ARGs and BMRGs was transformed to binary form based on the presence and absence of the genes in the fecal samples. Correlation tests were done between each ARG and BMRG using the Pearson method to identify if there was co-occurrence between specific ARGs and BMRGs. The strong correlations (*P* < 0.0001) that were found (where the absolute value of the correlation coefficient was greater than 0.5) between ARGs and BMRGs were kept for visualizations.

The visualizations of alpha diversity, predominant classes, and comparisons of classes by time for each feeding system were programmed through the ggplot2 package (*v3.4.2*) [39]. The NMDS ordination plots were visualized using base R. Heatmaps for relative abundance of ARGs were created using the ComplexHeatmap package (*v2.16.0*) [40]. The normalized relative abundance of ARGs was further normalized by row using the Euclidean method through the wordspace package (*v0.2–8*) [41] for better visualization. The transferable ARGs of each feeding systems, regardless of age, were visualized using the VennDiagram package (*v1.7.3*) [42]. The co-occurrence analysis was visualized using igraph (*v1.5.*0) [43] and ggraph package (*v2.1.0*) [44].

## Results

### Fecal resistome differs between feeding systems

**Fig. 1:**
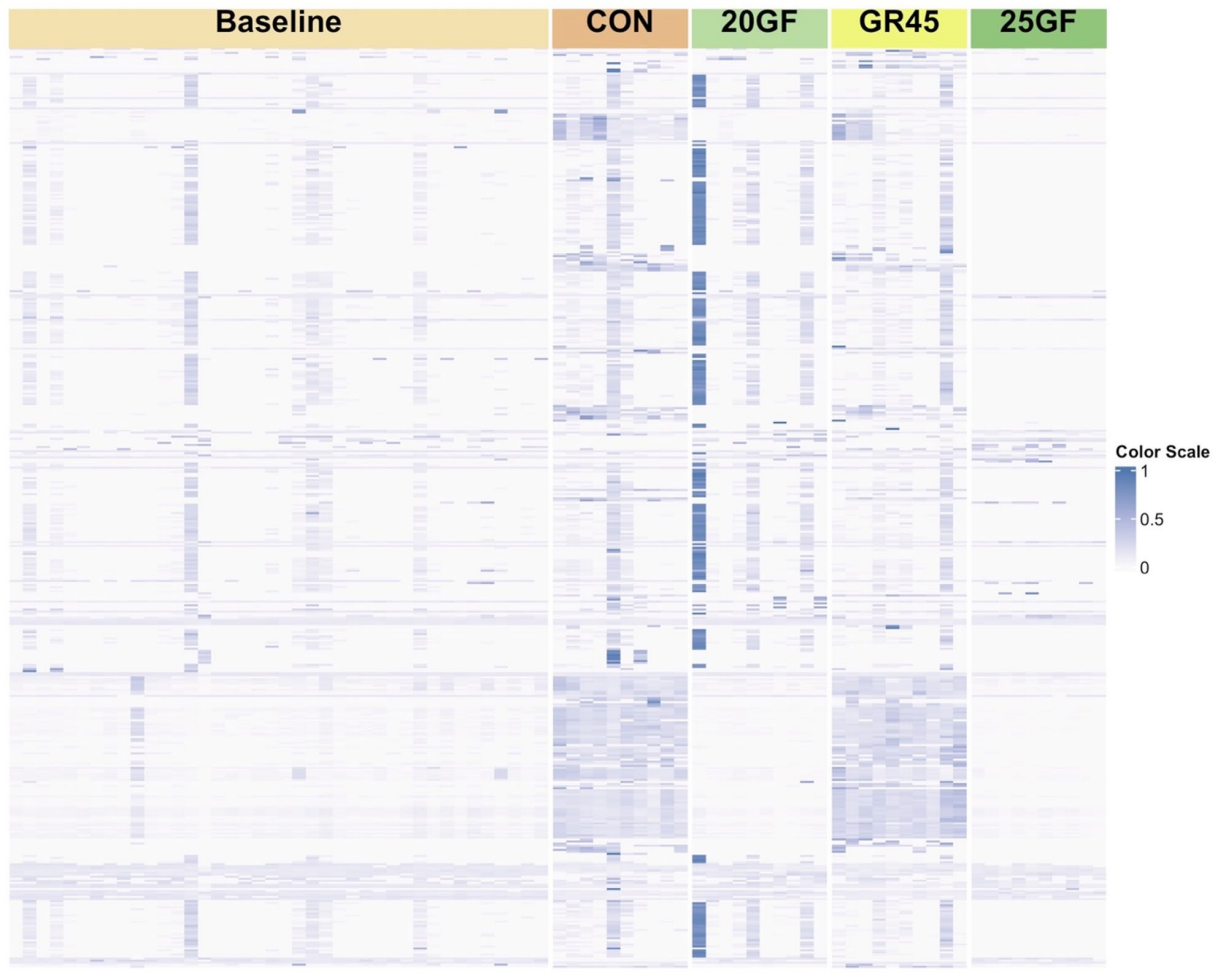
Heatmap of antimicrobial resistant genes (ARGs) identified in fecal samples collected at baseline combining four groups (*n*=40) and harvest for conventional grain-fed cattle (CON; *n *= 10), 20 months grass-fed cattle (20GF; *n *= 10), 20 months grass-fed and 45 days grain-finished cattle (GR45; *n* = 10), and 25 months grass-fed cattle (25GF; *n* = 10)

Over 41 million reads were kept after filtration, and these reads were aligned to 448 ARGs categorized into 40 classes of resistance and 89 mechanisms, with 341 ARGs identified across all groups. The average number of ARGs varied among groups, with the highest number identified in the CON (203, range from 74 to 440), followed by GR45 (185, range from 90 to 412), 20GF (158, range from 72 to 377), and 25GF (119, range from 66 to 373). A total of 132 out of 448 ARGs were identified across all groups at baseline. For the samples collected at harvest, 233 ARGs were identified in CON samples, 222 ARGs in GR45 samples, 164 ARGs in 20GF samples, and 88 ARGs in 25GF samples (Fig. 1).

**Fig. 2:**
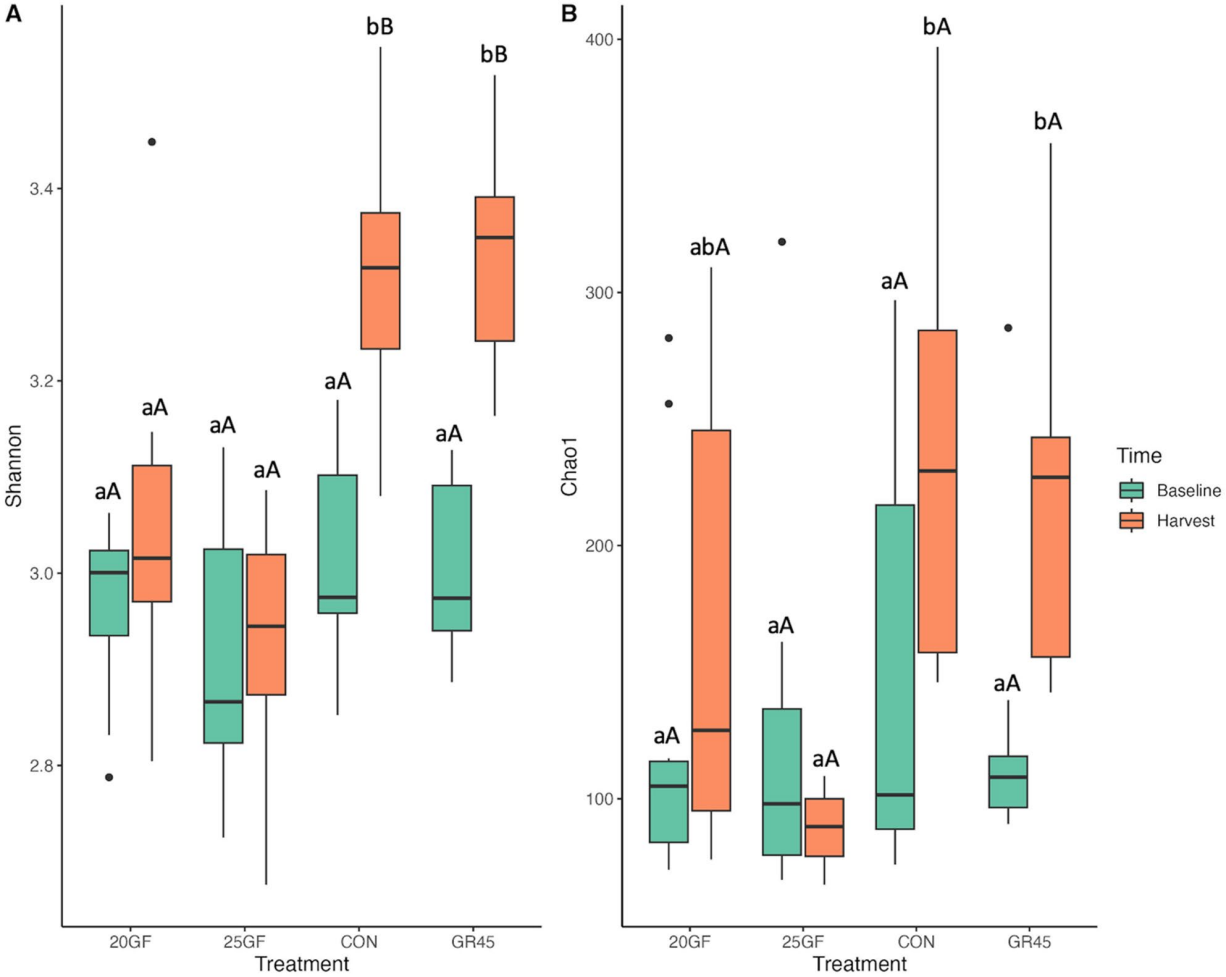
The comparisons of alpha diversity of microbial resistome at gene level of feces collected from cattle raised on different feeding systems (CON: Conventional grain-fed; 20GF: Grass-fed for 20 months, GR45: Grass-fed and then grain-finished for 45 days; 25GF: Grass-fed for 25 months). Superscripts a and b indicates the effects among feeding system at *P* < 0.05. Superscripts A and B indicate the difference between time of sample collection (baseline vs harvest) within the same feeding system. Superscripts a and b indicate the difference between different feeding systems (CON vs 20GF vs GR45 vs 25GF) at the time of the same sample collection (baseline or harvest). (**A**). Shannon diversity indexes of CON and GR45 increased from baseline to harvest, and the Shannon diversity of 20GF and 25GF were lower than CON and GR45. (**B**). Chao1 diversity indexes were higher in CON and GR45 compared with that of 25GF

At the gene level, the baseline alpha diversity, as determined by the Shannon and Chao1 indices, did not (*P* = 1.00) exhibit a difference across all groups. The Shannon index for grain-finished groups was higher (*P* ≤ 0.001) than for grass-feeding groups at the time of harvest (Fig. 2A). However, CON (3.32 ± 0.045) and GR45 (3.33 ± 0.037) had similar Shannon indexes (*P* = 1.00), and 20GF (3.05 ± 0.055) and 25GF (2.93 ± 0.04) also showed similar Shannon indexes (*P* = 0.49) (Fig. 2A). Notably, for 20GF (3.0 ± 0.03 to 3.0 ± 0.05 for Shannon from baseline to harvest; 129.7 ± 23.77 to 163.8 ± 28.24 for Chao1) and 25GF (2.0 ± 0.041 to 2.9 ± 0.04 for Shannon from baseline to harvest; 123.5 ± 23.88 to 88.3 ± 4.73 for Chao1), fecal microbial alpha diversity did not (*P* > 0.05) change in samples collected at the baseline and the harvest. Both the Shannon index (*P* < 0.001) and Chao1 index (*P* = 0.017) exhibited an increase as the cattle under GR45 group (3.01 ± 0.028 to 3.33 ± 0.037 of Shannon; 125.4 ± 18.43 to 222 ± 22.75 of Chao1) advanced in age (Fig. 2A and B). The CON group also displayed an increase in (*P* < 0.001) the Shannon index for the fecal microbes over time (3.01 ± 0.034 to 3.32 ± 0.045; Fig. 2A). Extensive grass-feeding resulted in a lower Chao1 index for 25GF (88.3 ± 4.73) compared with CON (233.2 ± 25.75; *P* = 0.0004) and GR45 (222 ± 22.75; *P* = 0.0014) at harvest, while 20GF (163.8 ± 28.24), CON, and GR45 had similar (*P* ≥ 0.45) Chao1 indexes (Fig. 2B). The findings indicate that fecal bacteria from the 25GF cattle had a lower number of ARGs, and compared with CON and GR45 these ARGs of both 20GF and 25GF were less evenly distributed among the samples in this group.

**Fig. 3:**
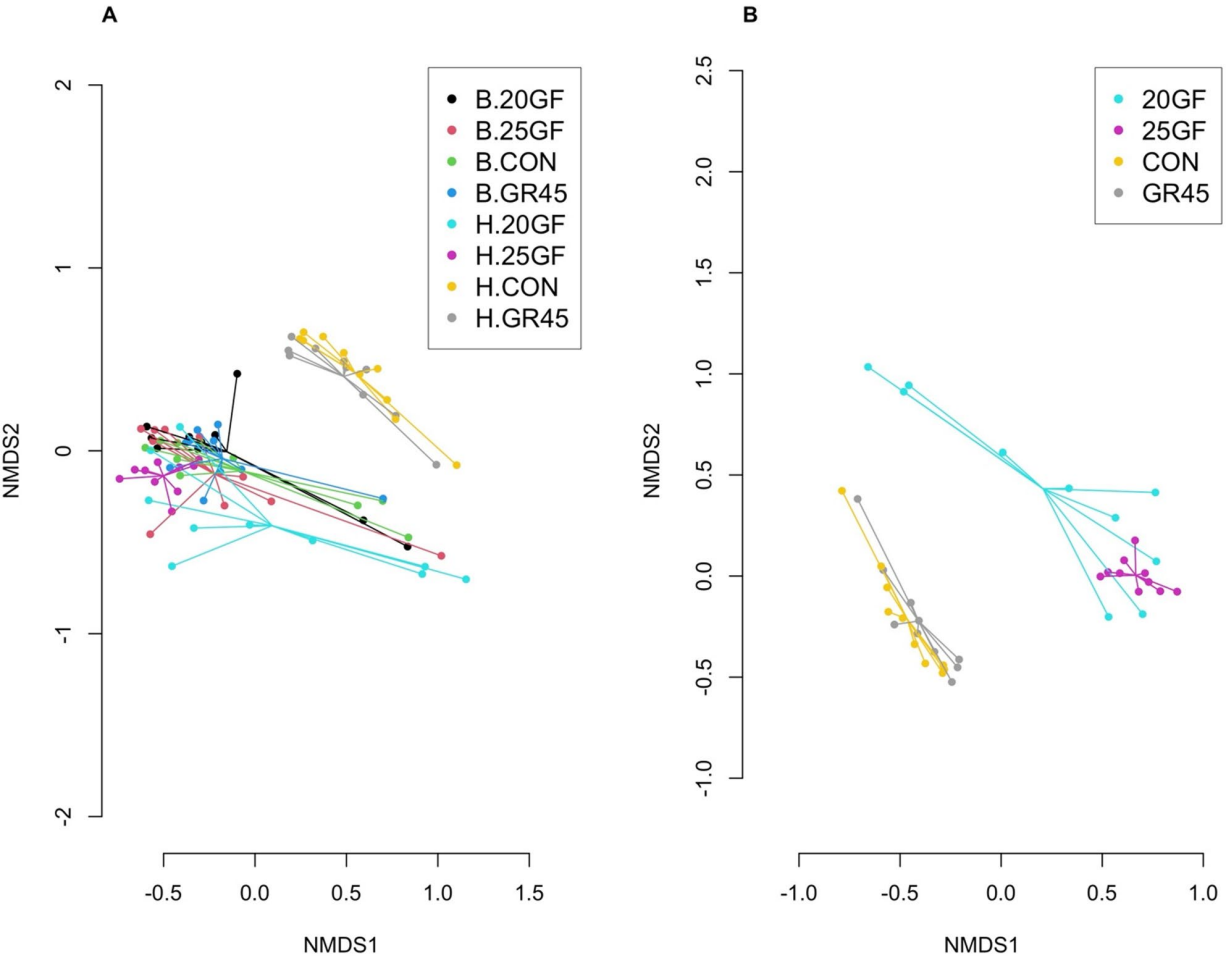
A comparison of fecal bacterial resistome as shown by non-metric multidimensional scaling (NMDS) ordination at gene level from cattle raised under different feeding systems (CON: conventional grain-fed; 20GF: grass-fed for 20 months; GR45: grass-fed and grain-finished for 45 days; 25GF: grass-fed for 25 months). (**A**). The four groups at two sampling time. “B” represents samples collected at baseline and “H” represents samples collected at harvest. CON at harvest (H.CON) and GR45 at harvest (H.GR45) were clustered apart other samples (ANOSIM (Analysis of Similarity) results: Stress = 0.07, ANOSIMR = 0.211, ANOSIM *P* = 0.018). (**B**). Microbial resistome of feces collected from cattle raised under four feeding systems at harvest (ANOSIM results: Stress = 0.06, ANOSIMR= 0.403, ANOSIM *P*= 0.001). 25GF resistome is different from of CON (*R*^2^= 0.66; *P* = 0.006) and GR45 (*R*^2^ = 0.50; *P*= 0.006), while the resistome of 20GF, CON and GR45 did not (*P* ≥ 0.054) show differences at harvest

The fecal resistome differed (Stress = 0.07, ANOSIM *R* = 0.211, ANOSIM *P* = 0.018) between CON and GR45 cattle groups and grass-fed cattle groups, regardless of the time of sample collection (Fig. 3A). When considering harvest samples, the difference led by feeding effects was more evident, showing a difference (Stress = 0.06, ANOSIM *R* = 0.403, ANOSIM *P* = 0.001) in beta diversity of different groups (Fig. 3B). Specifically, the resistome of 25GF was different from the resistome of CON (*R*^*2*^ = 0.66; *P* = 0.006) and GR45 (*R*^*2*^ = 0.50; *P* = 0.006), while the resistome of 20GF, CON and GR45 did not (*P* ≥ 0.054) show differences. The results demonstrate that different feeding systems give rise to distinct fecal resistome in cattle. Notably, at harvest, there is no statistical significance (*R²* = 0.096; *P* = 0.187) in the fecal resistome between CON cattle, receiving only monensin, and GR45 cattle, receiving both antibiotic drug in their lifetime and monensin for 45 days, suggesting that use of monensin also contributes to reshaping the fecal resistome. However, it is noteworthy that the duration of grazing serves as a critical factor in shaping the resistome within various grass-feeding systems. Cattle from similar feeding systems had a similar composition of ARGs in their feces, but the variance may vary based on antibiotics application and animal age. Taken the alpha diversity into consideration, the grain-finished groups enrich the diversity of the ARGs in cattle feces, whereas beef cattle raised under grass-feeding systems may harbor a low diversity of resistome.

**Fig. 4:**
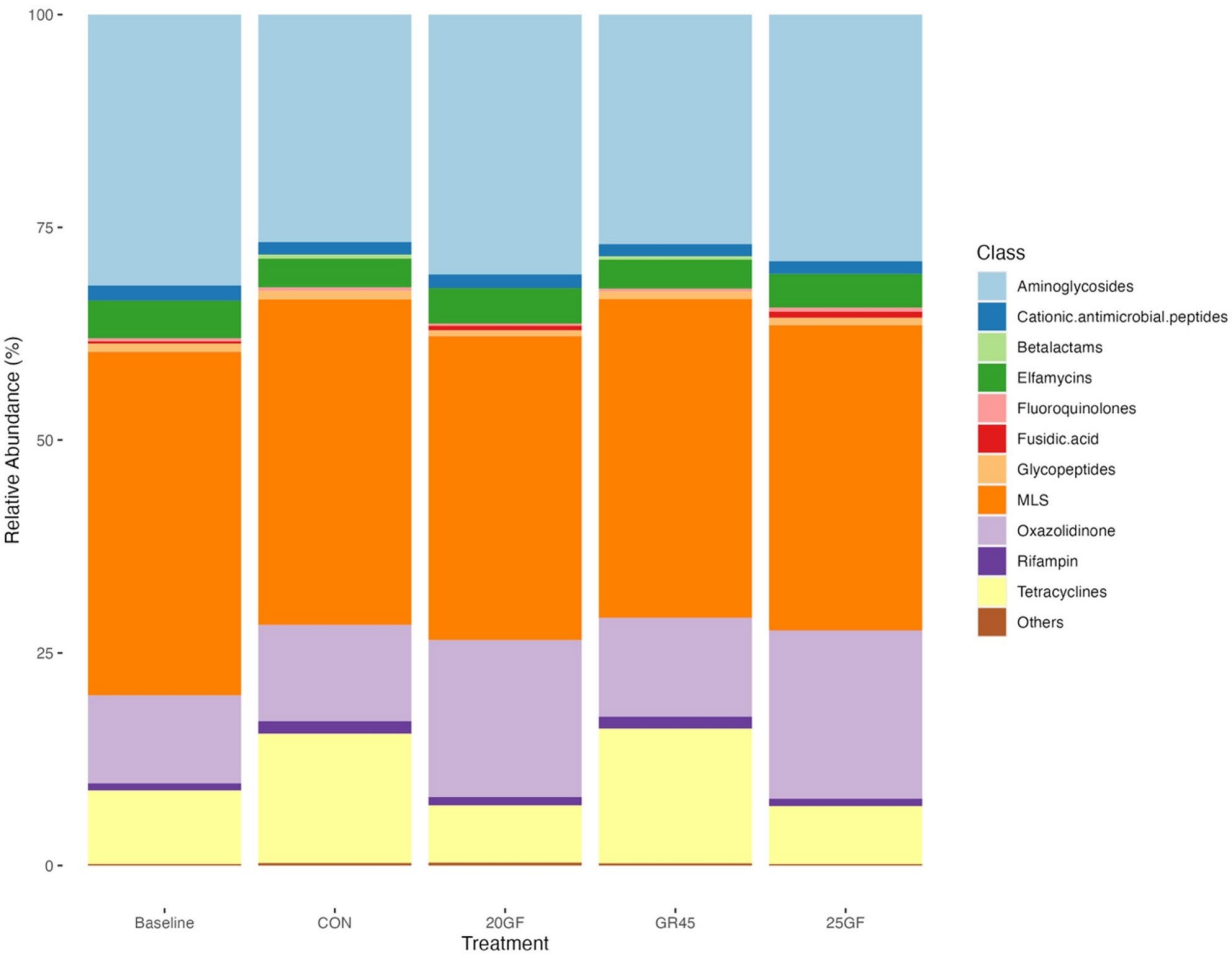
The relative abundance of top 10 antimicrobial resistant genes (ARGs) classes at baseline (*n*=40) and at harvest specifically for fecal bacteria collected from conventional grain-fed cattle (CON; *n *= 10), 20 months grass-fed cattle (20GF; *n* = 10), 20 months grass-fed and 45 days grain-finished cattle (GR45; *n* = 10), and 25 months grass-fed cattle (25GF; *n* = 10). Fecal samples collected from CON and GR45 showed similar composition of top 10 ARGs classes at harvest, while fecal samples collected from 20GF and 25GFshowed similar ARG composition

The top 3 most abundant classes of antibiotics to which ARGs encode resistance were macrolide-lincosamide-streptogramin (MLS), aminoglycosides, and oxazolidinone. Cattle raised under similar feeding systems had a similar composition and distribution of antibiotic classes in their feces. The relative abundance of tetracycline-associated ARGs in the CON (15.2%) and GR45 (15.8%) at harvest was higher (*P* < 0.0001) compared to both the baseline (8.66%) and grass-fed groups at harvest (6.72% for 20GF and 6.84% for 25GF, respectively). Fusidic acid, which was the 10th abundant class at the baseline, disappeared from the top ten classes for CON and GR45 cattle but became the 9th abundant class for 20GF and 25GF cattle, and a new class – betalactams – appeared in the top ten classes in CON and GR45 at harvest (Fig. 4).

**Fig. 5:**
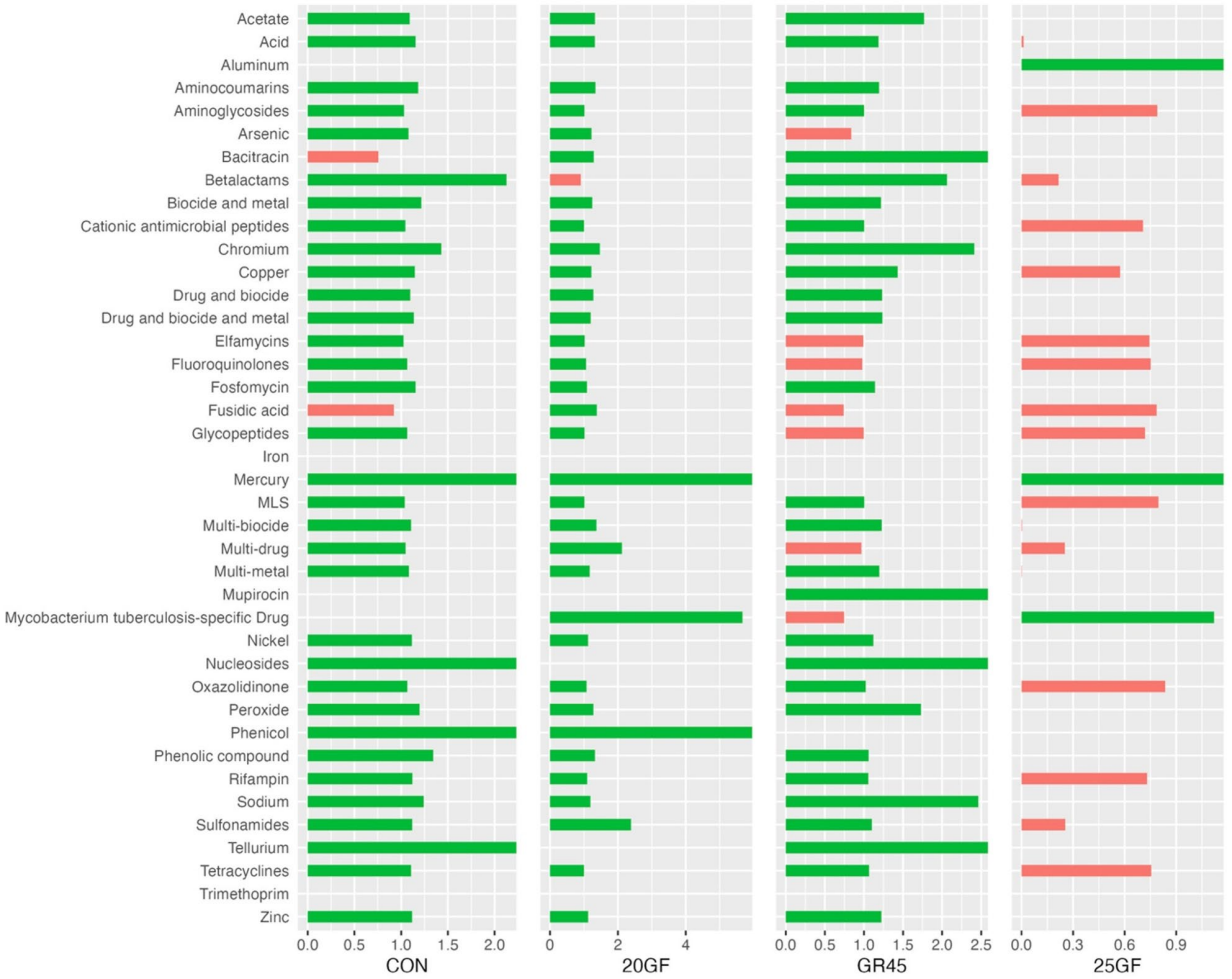
The relative abundance of antimicrobial resistant genes (ARGs) of fecal bacteria collected from conventional grain-fed cattle (CON; *n *= 10), 20 months grass-fed cattle (20GF; *n *= 10), 20 months grass-fed and 45 days grain-finished cattle (GR45; *n* = 10), and 25 months grass-fed cattle (25GF; *n* = 10) between baseline and harvest at class level. Red bar represents the reduction from baseline to harvest, while green bar represents the increment. 17 classes of ARGs no longer present at harvest of 25GF fecal bacteria, while overall trend of the relative abundance of cattle fecal ARGs from other groups inclined at harvest compared with the relative abundance at baselinecal bacteria, while overall trend of the relative abundance of cattle fecal ARGs from other groups inclined at harvest compared with the relative abundance at baseline

For CON, GR45, and 20GF, the overall trend of the relative abundance of reads aligned to drug classes inclined at harvest compared with the ones from the samples collected at baseline. However, the abundance of the reads to these drugs decreased for 25GF from baseline to harvest, with 17 of them disappearing at harvest (Fig. 5). Reads on the resistance to a handful drugs (such as nucleosides, phenicol, tellurium) were identified in samples collected only at the harvest (Fig. 5).

### Microbiome harboring ARGs is consistent between feeding systems

Overall, 19.5% of the reads were able to be classified into bacteria that shed the ARGs using RefSeq and RUG2 databases. *Pseudomonas*, *Escherichia*, *Klebsiella*, *Salmonella*, and *Synechocystis* were identified as the five most dominant genera for four groups, but the ranking was different in different groups (Table 1). No statistical significance (*P* ≥ 0.8) was observed in the relative abundance of these genera across the different feeding systems. *Pseudomonas* and *Escherichia*, as the top two most abundant genera across all groups, collectively constituted more than 11.6% of classable reads. The third most abundant genus was *Klebsiella* for both CON and GR45 groups, while *Synechocystis* occupied the same position for 20GF and 25GF groups. Notably, three out of the five most dominant genera belonged to the family *Enterobacteriaceae*, encompassing 10.3% of classable reads. The top five abundant bacteria species that shed ARGs were *Escherichia coli*, *Pseudomonas monteilii*, *Synechocystis sp. PCC 6803*, *Salmonella enterica*, and *Klebsiella pneumoniae*, ranked by order of abundance for 20GF, GR45, and 25GF, while the place of *Synechocystis sp. PCC 6803* and *Salmonella enterica* were switched for CON (Table 2). The relative abundance of either the same genus or the same species showed consistency (*P* ≥ 0.13) among different groups, although some numerical differences were observed. Overall, the findings indicate that the relative abundance of specific bacteria harboring ARGs remained consistent at both the genus and species levels across different feeding systems. However, it is notable that the feeding systems might exert a slight influence on the composition of the predominant genus and species within each respective feeding system.

**Table 1:** The relative abundance of the top five (out of 1781 total) predominant genera of fecal bacteria that shed antimicrobial resistant genes (ARGs) in fecal samples

	Groups^1^				
Genus	CON	20GF	GR45	25GF	*P*
Pseudomonas	1.81%	1.92%	1.84%	1.91%	0.99
Escherichia	1.61%	1.47%	1.61%	1.43%	0.98
Klebsiella	0.83%	0.69%	0.82%	0.69%	0.88
Salmonella	0.75%	0.67%	0.74%	0.65%	0.97
Synechocystis	0.72%	0.89%	0.74%	0.85%	0.80

^1^Groups: conventional grain-fed beef (CON), 20 months grass-fed beef (20GF), 20 months grass-fed and 45 days grain-finished beef (GR45), 25 months grass-fed beef (25GF). The relative abundance of each of the top five genera was the same when compared between different groups

**Table 2:** The relative abundance of top five (out of 7,705) predominant species of fecal bacteria that shed antimicrobial resistant genes (ARGs) in fecal samples

	Groups^1^				
Species	CON	20GF	GR45	25GF	*P*
*Escherichia coli*	1.58%	1.44%	1.40%	1.58%	0.26
*Pseudomonas monteilii*	0.87%	1.07%	1.04%	0.91%	0.21
*Salmonella enterica*	0.73%	0.66%	0.65%	0.73%	0.21
*Synechocystis sp. PCC 6803*	0.71%	0.89%	0.84%	0.74%	0.16
*Klebsiella pneumoniae*	0.69%	0.58%	0.59%	0.69%	0.13

^1^Groups: conventional grain-fed beef (CON), 20 months grass-fed beef (20GF), 20 months grass-fed and 45 days grain-finished beef (GR45), 25 months grass-fed beef (25GF). The relative abundance of each of the top five species was the same when compared between different groups

### Feeding systems lead to different numbers of transferable ARGs

**Fig. 6:**
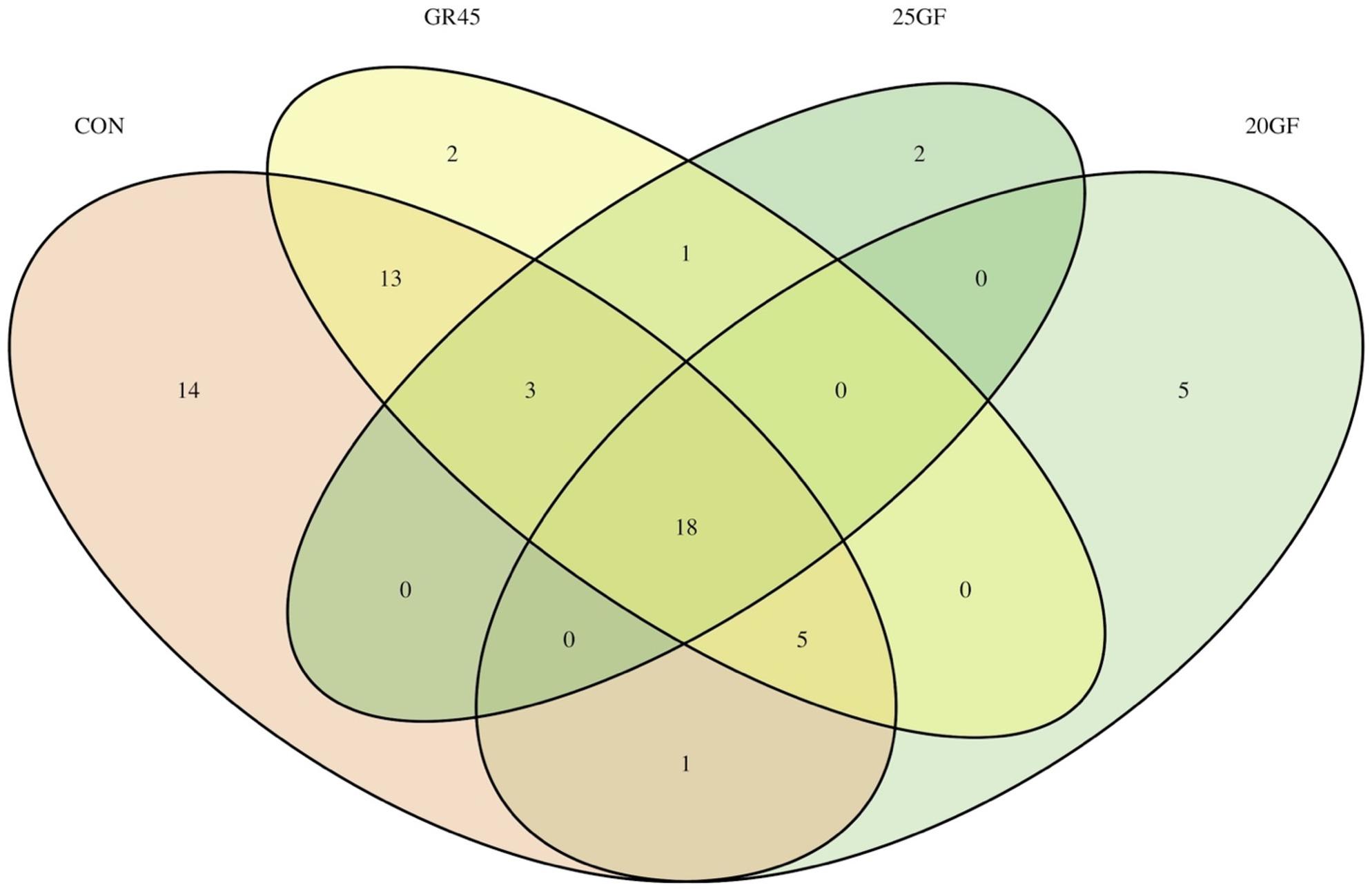
Transferable antimicrobial resistant genes (ARGs located on plasmid and transposon) identified in the fecal bacteria of conventional grain-fed cattle (CON; *n* = 10), 20 months grass-fed cattle (20GF; *n* = 10), 20 months grass-fed and 45 days grain-finished cattle (GR45; *n* = 10), and 25 months grass-fed cattle (25GF; *n* = 10) regardless of age. CON (54) showed the most transferable ARGs, followed by GR45 (42), 20GF (29), and 25GF (24)

The number of transferable ARGs identified for CON, 20GF, GR45, and 25GF were 54, 29, 42, and 24, respectively, while the number of unique transferable ARGs identified was 14, 5, 2, and 2, respectively. Eighteen common transferable ARGs were presented in feces from cattle in all groups (Fig. 6). Among the common transferable ARGs, eight of them exhibited resistance to tetracycline, two were resistant to beta-lactam, two were resistant to lincosamide, and two showed resistances to chloramphenicol-florfenicol.

### Biocide and metal resistome did not differ among feeding systems

**Fig. 7:**
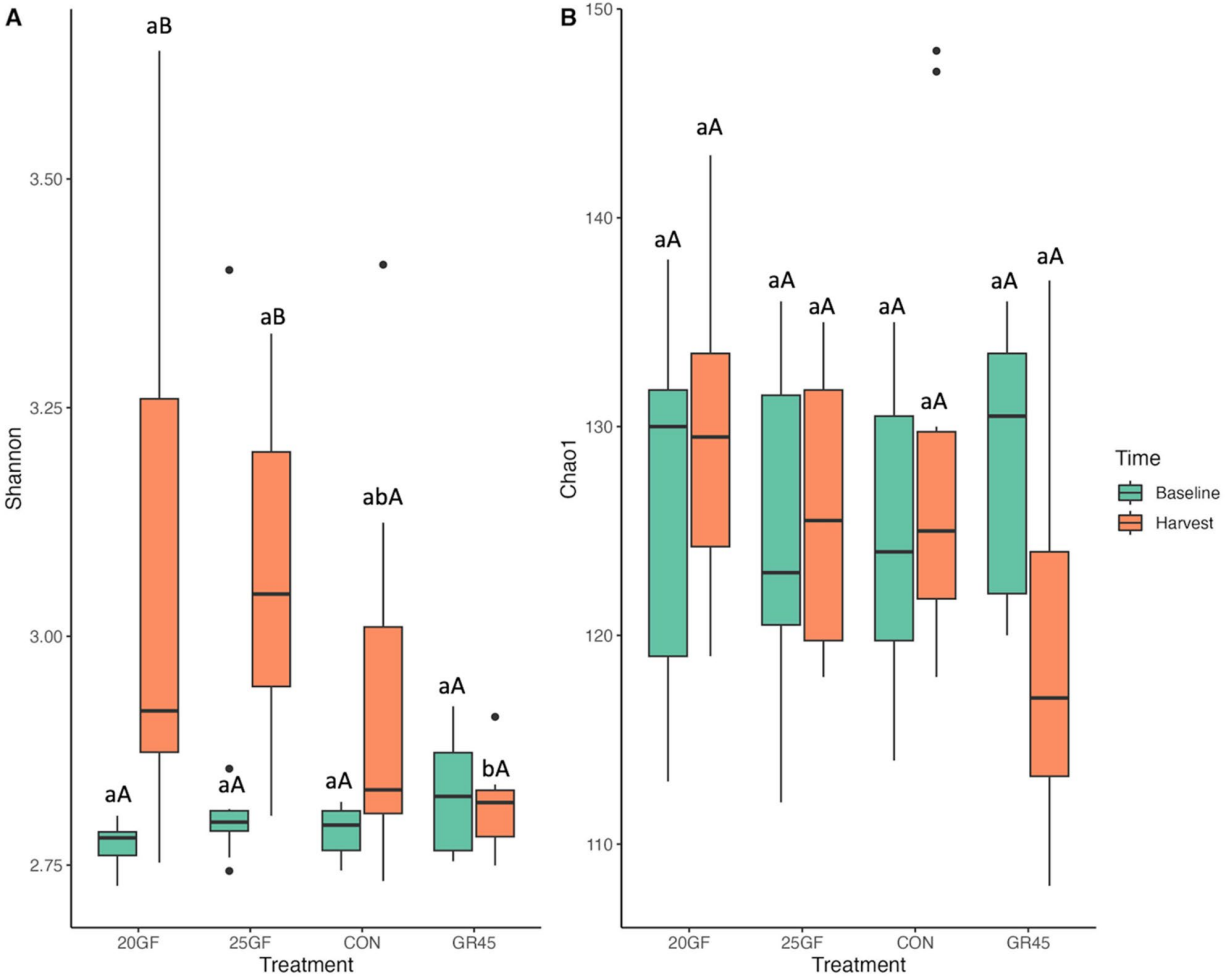
The comparisons of alpha diversity of biocide and metal resistant genes located on plasmid and transposon (PTBMRGs) at gene level of feces collected from cattle raised on different feeding systems (CON: Conventional grain-fed; 20GF: Grass-fed for 20 months, GR45: Grass-fed and then grain-finished for 45 days; 25GF: Grass-fed for 25 months). Superscripts a and b show the difference among feeding groups CON, 20GF, GR45, and 25GF at the same time of sample collection. Superscripts A and B indicate the difference between time of sample collection (baseline vs harvest) within the same feeding system. Superscripts a and b indicate the difference between different feeding systems (CON vs 20GF vs GR45 vs 25GF) at the time of the same sample collection (baseline or harvest). (**A**). Shannon diversity indexes were increased in 20GF and 25GF from baseline to harvest. (**B**). Chao1 diversity indexes were all the same regardless of sampling time and groups

**Fig. 8:**
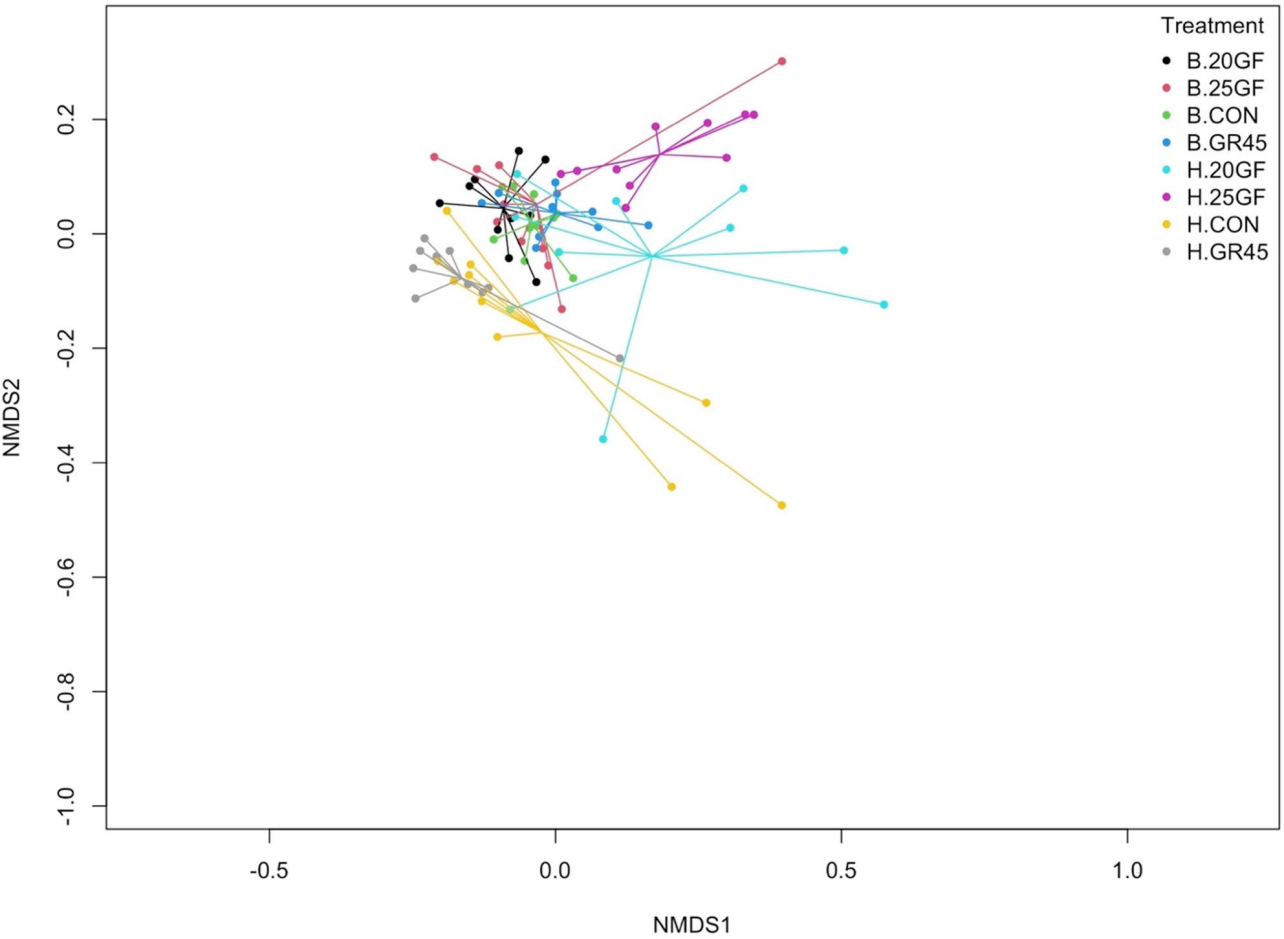
A comparison of fecal samples revealing by non-metric multidimensional scaling (NMDS) ordination of biocide and metal resistome at gene level from four cattle raised under different feeding systems (CON: conventional grain-fed; 20GF: grass-fed for 20 months; GR45: grass-fed and grain-finished for 45 days; 25GF: grass-fed for 25 months) at two sampling time (Stress= 0.14, ANOSIM R = 0.20, ANOSIM *P* = 0.16). “B” represents samples collected at baseline and “H” represents samples collected at harvest. Samples were not clustered by either groups or sampling time

Out of the total 753 BMRGs identified, 170 of them were confirmed to be located on plasmid and transposon (PTBMRGs), which were selected for further analysis. No statistical significance (*P* = 0.45) was detected in Chao index for the biocide and metal resistome of microbes from feces collected from cattle raised among different feeding systems. However, a lower Shannon index (2.82) was found for GR45 cattle comparing with those for 20GF (2.92; *P* = 0.0093) and 25GF (2.96; *P* = 0.013)) cattle (Fig. 7). Beta diversity of PTBMGRs did not differ (Stress = 0.14, ANOSIM *R* = 0.20, ANOSIM *P* = 0.16) among all the groups (Fig. 8), indicating that the investigated feeding systems in the present study did not influence the biocide and metal resistome in cattle feces. This lack of difference may be attributed to the prior mineral supplementation provided to all groups.

### ARGs are co-occurrent with PTBMRGs

**Fig. 9:**
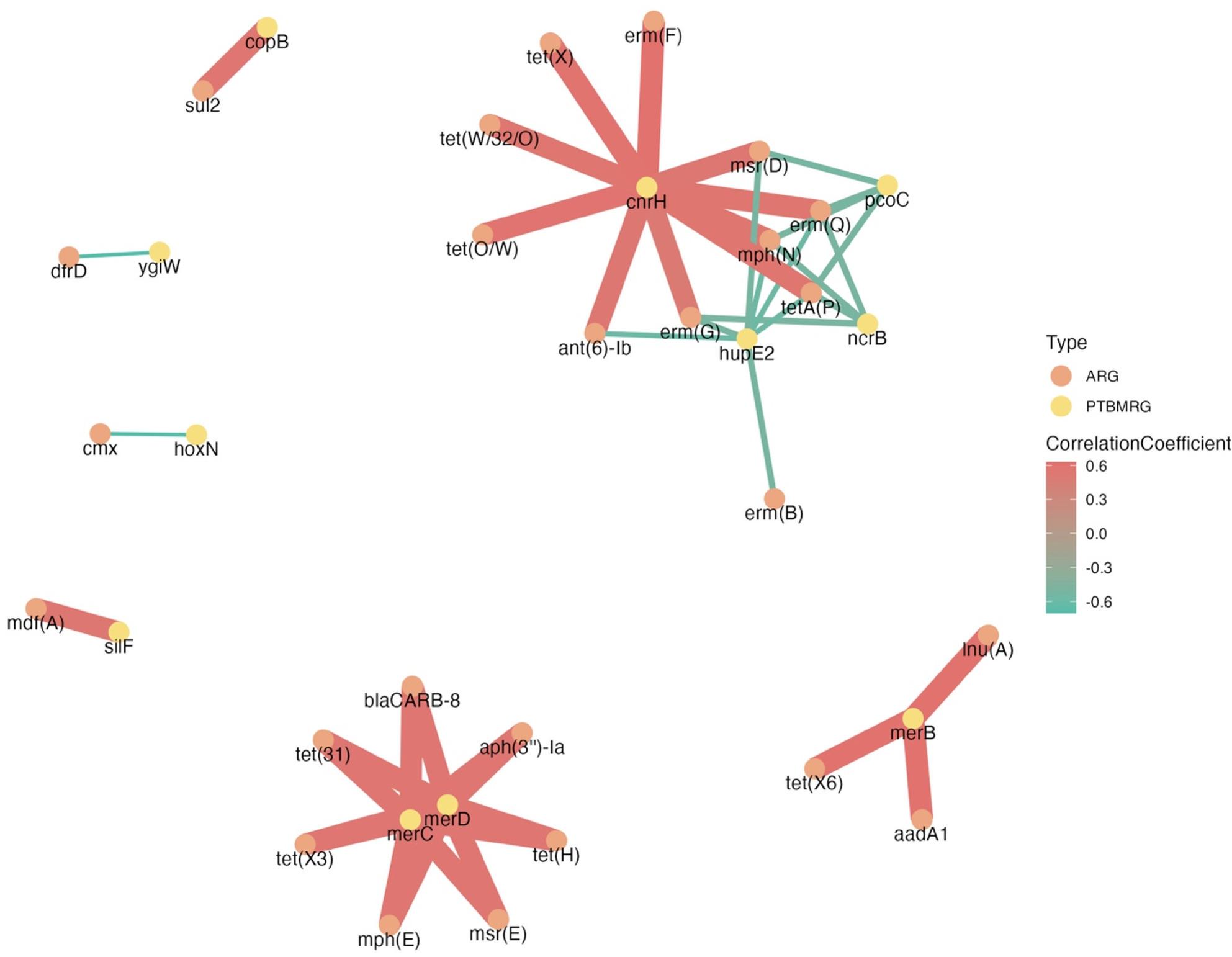
The co-occurrence analysis for antimicrobial resistant genes (ARGs) and biocide and metal resistant genes locating on plasmid and transposon (PTBMRGs) revealed four clusters centered around various PTBMRGs, with three exhibiting positively correlated (*r* >0.5; *P* < 0.0001) connections and one displaying a negatively correlated (*r* >0.5; *P* < 0.0001) connection

Among 3717 valid correlation tests, 46 of them were strong correlations (*r* > 0.5; *P* < 0.0001). The results indicated that one PTBMRG could positively or negatively correlate with multiple ARGs. For example, the presence of *merC* and *merD*, which are resistant to mercury, were strongly positively correlated (*P* < 0.001) with the presence of seven ARGs, which are resistant to tetracycline (3), beta-lactam (1), macrolide-lincosamide-streptogramin (1), aminoglycoside (1), and macrolide (1) (Fig. 9). The presence of *cnrH* with resistance to cobalt and nickel was also strongly positively correlated (*P* < 0.001) with the presence of 10 ARGs, including phenotypes of macrolide resistance (4), tetracycline resistance (4), aminoglycoside resistance (1), and macrolide-lincosamide-streptogramin resistance (1) (Fig. 9). (Fig. 9). Interestingly, The occurrences of *hupE2* (nickel resistance), *pcoC* (copper resistance), and *ncrB* (cobalt and nickel resistance) decreased (*P* < 0.001) the probability of the presence of seven ARGs, four ARGs, and four ARGs independently (Fig. 9). The presence of two copper resistant PTBMRGs was strongly correlated with ARGs, but one (*copB*) was positively correlated, and one (*pcoC*) was negatively correlated. Among the five PTBMRGs having a negative correlation with ARGs, three of them (*ncrB*, *hupE2*, and *hoxN*) were nickel (Ni) resistant.

## Discussion

This study demonstrated that grass-feeding systems, especially extended grass-feeding (25GF), had a less diverse resistome, suggesting that monensin use and the feedlot environment are key drivers of resistome diversification, as shown by the rapid shift in GR45. Cattle raised in grass-feeding systems exhibited lower richness and evenness of ARGs in their feces than cattle raised in the conventional grain-finished feeding systems that incorporated monensin in the feed, as indicated by the Shannon and Chao1 indices. The long-term use of monensin, a well-known antibacterial ionophore, and/or therapeutic antibiotics in grain-feeding systems may contribute to the development of resistance or reshape the resistome in fecal bacteria. Previous research has suggested that monensin exerts selective pressure on gram-positive cells, leading to alterations in the growth of *Enterococcus faecium* and *Enterococcus faecalis* in the pure culture, potentially influencing resistance to ARGs [45]. *Enterococcus* has been detected across all the groups in this study; however, it accounts for 0.19% of the ARG reads in the fecal samples of cattle treated with monensin and therapeutic antibiotics. Therefore, *Enterococcus* may not significantly contribute to the observed AMR patterns in this investigation. Holman et al. [46] found that a single injection of oxytetracycline altered the fecal microbiota and enriched ARGs in cattle, compared to cattle that had not received any antibiotics. Similar findings were reported in another study conducted by Rovira et al. [47], where it was observed that the number of ARGs identified in the conventional cattle feeding system (beef and dairy) was 51% higher than in feeding systems without antibiotic administration after excluding ARGs found in wastewater. Similar to the current study, Rovira et al. [47] considered antibiotic administration and assessed environmental factors such as water sources when evaluating AMR in cattle from different feeding systems. Unlike our study, where baseline samples were collected prior to assigning cattle to groups, Rovira et al. [47] collected fecal samples during the early feeding period (13 ± 11 days) and late feeding period (243 ± 48 days) in the feedlot. Nevertheless, both studies yielded similar results that the fecal resistome remained consistent among groups during the early period in their study and at baseline in this study, indicating that resistome transforms after a few days of switching feeding systems. Our study showed that the composition of the top ten ARG classes of GR45 was closer to that of CON than that of 20GF. The quick change in the fecal resistome of GR45 cattle towards the cattle fecal resistome in the conventional feeding system can be attributed to the 45-day finishing period in the feedlot. Compared to the 20GF resistome, the composition of ARG classes in GR45 is much closer to that of CON, which indicates that the conventional feedlot, which usually supplements monensin in feed ration along with therapeutic antibiotics, has a powerful potential to restructure the resistome within a short-time period. While antibiotic use appears to be a key driver of resistome alteration, other factors including diet composition, environment, and cattle age likely contributed to the observed differences. These confounding factors should be considered when interpreting the relationship between antibiotic exposure and ARG diversity.

Environmental factors could also have played a role in shifting the resistome. Feedlots, which are used to enhance the efficiency of beef production, are known for their dense stocking conditions where cattle typically congregate around feed and water areas, and a notable presence of ARGs has been detected around feed and water sources in the feedlot [48]. Water and feed can become contaminated by animal feces, facilitating the spread of AMR through horizontal gene transfer of ARGs among bacteria within these environmental sectors [48]. A study conducted by Agga et al. [48] demonstrated that the concentration of ARGs specifically related to sulfonamide is the highest in the proximity of waterers and feeders, while it is lowest in grazing areas located farthest from the feeding area. Therefore, the different fecal resistome among cattle under different feeding systems can be attributed to the higher stocking densities of cattle in conventional systems, where they are often confined to close living quarters. It is also possible that beef cattle from CON and GR45 gained more ARGs from their environment, such as soil, and then ARGs were further transmitted to others via close interaction, including the sharing of feeders and water troughs. In contrast, grass-feeding systems allow cattle to roam and graze on pastures, which may limit the transmission and acquisition of ARGs. This theory can also explain the higher detection of transferable ARGs in CON, followed by GR45, 20GF, and 25GF. The number of transferable ARGs could increase dramatically within 45 days for GR45 in the feedlot, as the intensive population in feedlots creates an ideal environment for ARG transfer. Additionally, Shawver [49] revealed that soil samples exposed to manure from cattle, including both antibiotic-free and antibiotic-treated cattle, exhibited a high abundance of ARGs belonging to tetracycline, aminoglycoside, and sulfonamide classes. According to the annual summary report on antimicrobials sold or distributed for use in food-producing animals by the FDA [50], 45% of sulfonamides, 52% of aminoglycosides, and 43% of tetracyclines were intended for medical use in cattle in 2021. A significant proportion of administered antimicrobial substances, ranging from 20% to 80%, is excreted in the form of metabolites through urine and feces, which can persist in the soil even after the animals have been removed [48]. Therefore, when antibiotics were previously administered to cattle within the same feedlot, residual traces of these antibiotics could be excreted by the cattle into the soil. The dispersion of antibiotics, accompanied by flying dust, could potentially lead to the contamination of feed or water, facilitated by cattle movement. The hypothesis that ARGs present in the animals’ living environment can colonize in the animals even without direct exposure is further supported by other studies [51, 52].

In this study, the results revealed that age could contribute to altering resistome abundance. For 25GF, 42.5% (17 classes) of ARG classes showed reductions at harvest, and 42.5% (17 classes) of ARG classes were present at baseline but disappeared at harvest, while 7.5% (3 classes) increased in prevalence from baseline to harvest. The cattle in 20GF, which received the grass diet but were harvested earlier, did not exhibit a significant decrease in the prevalence of ARGs from the baseline to the harvest stage. This lack of reduction could be attributed to the additional five months of grazing on irrigated pasture or to the older harvest age for 25GF cattle. This idea is supported by previous research that the prevalence of ARGs in fecal samples decreased in aged beef cattle, dairy cattle, and swine [53]. In the present study, the 25GF cattle exhibited the greatest ARG prevalence reduction in their feces at harvest. However, this does not imply that older animals always harbor fewer ARGs, as there may be a significant increase in AMR development after reaching a certain age. According to Robey et al. [54], bacterial resistance to amoxicillin, cefalexin, ciprofloxacin, co-amoxiclav, and extended-spectrum beta-lactamases (ESBL)-producing *E. coli* decreases (amoxicillin, ciprofloxacin, and co-amoxiclav) or remains stable (cefalexin) during early ages of humans, and then increases as individuals grow older. In humans, specific change points for amoxicillin, cefalexin, ciprofloxacin, and co-amoxiclav are observed at 12 years, 54 years, two years, and 33 years, respectively [54]. These findings, along with previous studies, suggested that the age-dependent dynamics of ARG fluctuations vary across animal species and types/classes of antibiotics. Further research is necessary to determine the age at which cattle harbor the lowest abundance of ARGs to minimize the transmission of AMR. Nevertheless, in this study, age and feeding systems could potentially serve as confounding factors. The cattle in the 25GF group are not only the oldest among the groups but also have not been directly exposed to antibiotics for an extended duration. This lack of direct antibiotic exposure could also be a contributing factor that led to the lower abundance of ARGs observed in this particular group.

The most abundant class of ARGs in the fecal samples was MLS, followed by aminoglycosides, oxazolidinone, or tetracyclines, regardless of the feeding system and age of the animals. Similar results have been shown in earlier studies where the resistance of MLS, aminoglycosides, and tetracyclines were all observed in greater than 50% of the samples from cattle [16, 22, 55]. In the present study, more than 70% of ARGs belong to these three classes regardless of groups, and greater than 80% of ARGs are categorized in these three classes for baseline and grain-feeding groups. Previous studies had shown that the therapeutic use of oxytetracycline could increase the minimal inhibitory concentration of tetracycline by encoding *tet*(A) and *tet*(B) in *Escherichia coli* (*E. coli)*, and the subcutaneous injection of oxytetracycline could traverse the biliary route and exert selection pressure on intestinal bacteria [56, 57]. Cattle from the GR45 group received oxytetracycline at different time points between June and October 2018. Fecal samples were collected in November, and it is difficult to trace the exact dosage and assess the therapeutic effect of the oxytetracycline. However, none of the cattle from the other feeding systems were directly exposed to oxytetracycline, which indicated that tetracycline-associated ARGs may be naturally present in the fecal bacteria of cattle. In contrast to another study [16], our research identified a significant occurrence of oxazolidinone resistance across all groups (the third most abundant class at baseline and for 20GF and 25GF at harvest; the fourth most abundant class for CON and GR45). It was important to note that while oxazolidinones were approved for clinical use in humans and companion animals, their use was never approved for food-producing animals in the United States [58]. A similar situation was reported that 30% of the poultry carried fluoroquinolone-resistant *E.coli* in Australia where fluoroquinolone is prohibited in food-producing animals [59]. Therefore, the presence of oxazolidinone resistance observed in the current study may have originated from contaminated wastewater. Linezolid has been detected in the urine of patients treated with the drug [60]. Wastewater containing linezolid may be used to irrigate pasture, potentially introducing resistance genes to livestock. Additionally, oxazolidinone resistance may have also arisen from human sources, such as workers involved in cattle production. Another potential source could be companion animals with access to these production systems, which may excrete feces with ARGs. This could facilitate horizontal gene transfer, where bacteria harboring resistance genes are transferred from companion animals to the soil and subsequently to cattle. Among the 18 common transferable ARGs investigated, 16 of them demonstrated resistance to tetracycline, beta-lactam, or lincosamide antibiotics, which are widely utilized in both veterinary and human medicine [61–63]. On the other hand, two of the ARGs showed resistance to chloramphenicol-florfenicol. Notably, florfenicol is restricted in human medicine due to its severe side effects but remains used for veterinary purposes concerning pets and food-producing animals [64]. Consequently, the concern about florfenicol-resistant genes alone in cattle fecal bacteria might not be overly significant for humans; however, co-selection mechanisms between florfenicol-resistant genes and other classes of resistant genes could pose a potential risk. Furthermore, chloramphenicol is approved for treating infections in pets but is considered illegal for use in cattle and pigs and is only used for serious human diseases [65]. Thus, the presence of chloramphenicol resistant genes in animals from feedlot could be explained by close contact with farm workers and possibly pets. In addition, soil bacteria naturally produce chloramphenicol, which could expose cattle on pasture to the antibiotic and lead to the development of resistance [66]. Further research is needed to identify the transmission routes from various sectors on the farm (e.g., people, pets, wild animals, and soil). Meanwhile, the results of this study are based on sequencing data, which cannot fully reflect the phenotypic traits of the fecal bacteria. Therefore, further studies should be conducted to analyze phenotypic data, providing a more accurate assessment of the severity and potential risks of AMR.

In our study, *Enterobacteriaceae*, including *Escherichia*, *Klebsiella*, and *Salmonella*, emerged as the predominant family that shed ARGs across all groups. *Enterobacteriaceae* has been exhibited to produce 16 S rRNA methyltransferase, which is a high-level resistance mechanism against aminoglycosides [67]. Therefore, *Enterobacteriaceae* has naturally developed resistance against aminoglycoside. *Escherichia* and *Klebsiella* are the two predominant bacterial genera that shed ARGs in this study. These genera are known to be primary hosts for multidrug-resistant genes [68], which might be the result of the naturally occurring resistant mechanisms conferring cross-resistance to multiple antibiotic classes. Coque et al. [69] demonstrated that ESBL-producing *Enterobacteriaceae* develop resistance to beta-lactam antibiotics and co-select ARGs for fluoroquinolones or aminoglycosides. In our study, ESBL-producing *Enterobacteriaceae*, including *Escherichia coli* and *Klebsiella pneumoniae*, emerged as the most and fifth abundant bacteria species responsible for harboring ARGs across all groups. The inherent capability of producing ESBL renders these bacteria resistant to beta-lactam antibiotics, and the high abundance of aminoglycoside and fluoroquinolone resistant genes may be attributed to co-selection mechanisms. Of significance, the relative abundance of the predominant genera and classes of bacteria harboring ARGs did not show significant variation between groups. This finding suggests that feeding systems may not influence certain bacterial ability to shed ARGs. Furthermore, the results emphasized again that studying the AMR patterns of a few bacterial species may inadequately capture the comprehensive AMR landscape of the entire system. The employment of metagenomics to investigate the resistome holds the potential to enhance the understanding of resistant genes and mechanisms, extending its efficacy to contain even those bacterial species that are not culturable [70].

The exposure of cattle feces to biocides and heavy metals has been shown to induce the development of BMRGs [71, 72]. Interestingly, in this study, no significant differences in the biocide and metal resistome were detected across the different groups. This lack of variation could be attributed to the fact that all cattle involved in the study had received mineral supplementation at Sierra Foothill Research and Extension Center before being assigned to their respective groups, although 20GF and 25GF cattle kept receiving the same mineral supplementation later for winter-spring grazing. However, a previous study yielded different results, indicating that the total abundance of BMRGs in grazing cattle feces was notably lower than in intensive feeding cattle feces [73]. The disparity might arise from the fact that the grazing cattle in that study did not have direct exposure to any antimicrobials, biocides, or heavy metals. Furthermore, all the feedlot cattle were provided with mineral supplementation in their feed ration in our study, which implies that the duration of exposure to minerals and biocides might not exert an influence on their resistome as long as direct exposure has occurred. Nonetheless, this hypothesis requires confirmation through future research.

The ARGs were co-selected with BMRGs, which could have been due to the close location of those genes or the same resistance mechanisms shared by those genes [74]. When ARGs and BMRGs are physically linked on the same mobile genetic elements (such as plasmids or transposons), exposure to one selective agent can simultaneously enrich for both resistance traits. This genetic linkage can facilitate the persistence of ARGs in environments where antibiotic use is limited but heavy metal contamination persists, such as soils supplemented with trace minerals or exposed to industrial runoff. Two of the three clusters were centered around the PTBMRGs showing resistance to mercury (Fig. 9). Mercury had been used against both eukaryotes and prokaryotes due to its antimicrobial characteristics [75], and the residues in the soil might have induced the development of mercury-resistant genes. The three mercury-resistant genes were strongly positively correlated with 10 ARGs, and four of them were tetracycline-resistant. A similar result had been shown phenotypically that 78.8% of mercury-resistant *Staphylococcus aureus* were resistant to tetracycline at 100 and 200 µg/ml, while only 44.7% of the mercury-sensitive *S. aureus* were resistant to the same concentrations of tetracycline [76]. The previous study [76] and the present study demonstrated both the phenotypical and genotypical co-occurrence of mercury-resistance and tetracycline-resistance. These findings may be explained by global industrialization, which has led to a twofold increase in mercury concentration in sediment and water. This rise corresponds to an increase in mercury-resistant bacterial genes in the same area [77]. Past studies also showed different effects of antibiotics on the growth performance of copper-resistant bacteria. The study done by Boyd et al. [78] indicated that copper-resistant *E. coli* grew slower in the presence of the antibiotics chloramphenicol, bacitracin, and sulfonamide class, which increased antibiotic susceptibility through a nonpharmacological approach. Another study showed contradictory results; the fecal *Enterococci* extracted from feedlot cattle containing the transferable copper-resistant gene were also resistant to tetracycline, tylosin, and vancomycin [79]. Considering copper is a common supplement in cattle diets for antimicrobial purposes, more research has to be conducted to confirm whether copper can be a safe antibiotic alternative without indirectly inducing the development of AMR. In our study, resistance to Ni was detected to be negatively correlated to some specific ARGs (such as *erm(Q)*, *tetA(P)*, *cmx*, etc.). Based on the study by Singh et al. [80] that 3.0 mg Ni/kg dry matter can improve the growth performance of cattle, nickel could be a potential antibiotic alternative by increasing animal performance while suppressing the occurrence of certain ARGs, but further research has to be done to investigate it.

## Conclusions

The findings of our study revealed a significant difference in the fecal resistome of cattle raised under conventional and grass-feeding systems. Overall, conventional feedlot cattle and grain-finished feedlot cattle exhibited a greater diversity of ARGs in their feces compared to those of 100% grass-fed cattle. The similarity in diversity and composition of ARGs between CON and GR45 suggests that even a short period of antibiotic administration can shift the fecal resistome of cattle. In conclusion, the predominant factors contributing to the shift of resistome in conventional feedlots are likely the use of antibiotics for disease prevention and treatment, in addition to other potential components of the production environment, such as geographic region, soil conditions, and interactions with other animals in the living niche. Further studies should be conducted to fully comprehend the independent effects of these components on the resistome shift in different feeding systems. Future research should also aim to identify the relative impacts of preventive use of antibiotics (e.g., monensin) versus therapeutic treatment, as well as evaluate the influence of short-term versus long-term exposures on the resistome.

Additionally, animal feeding duration appears to be a critical factor in reducing AMR in cattle feces, as evidenced by the lowest abundance of ARGs observed in 25GF cattle. Notably, after 20 months of grazing, the 25GF cattle were relocated to a different grazing site, highlighting the need for further research to determine whether this geographic change contributed to the observed differences in ARG abundance. Furthermore, while feeding duration may influence fecal AMR development, other factors, such as animal growth performance, market weight, meat quality, and cost of production, should also be considered when making practical decisions regarding feeding strategies.

## Data Availability

The datasets generated for this study can be found in the BioProject database http://www.ncbi.nlm.nih.gov/bioproject/1217999.
